# Description of the Inauguration of the Statue of Broussais

**Published:** 1842-06-18

**Authors:** 


### ANALECTA.

[We extract a description of the inauguration of the statue of Broussais from the last No. of the Medico-Chirurgical Review. The ceremony, it will be seen, was attended with so much pomp as almost to pass the boundaries of the sublime into the ridiculous.

The Secretary of the Academy of Medicine, M. Pariset, was extremely lukewarm in his eulogium. Begin, as military surgeon, and Bouillaud, as representative of the faculty, were the only orators who seemed at all enthusiastic in the matter. Professor Bouillaud's enthusiasm for the doctrines and the person of his teacher does not, however, meet much sympathy among his colleagues. They feel as every one feels, that Broussais was an active, daring genius, who left little or no additions of his own to science; but, by the impulse he gave to the researches of others, he has exercised the most happy indirect influence, more, we believe, than enough to overbalance any injurious effects from his exclusive doctrines. As a practical work, however, we would always except his first and best work on the Chronic Inflammations, as free from the imperfections of his writings, and full of fertile hints for a practical mind.]

"Three years have elapsed since the death of ***Broussais.*** The first of December, 1838, will ever be regarded as a great historical date. On that day science was struck in the head; that day beheld the extinction of one of the brightest intelligencies that have ever illumined the world; the nineteenth century suffered an irreparable loss, and France wept o'er one of her most glorious children."

The funeral, we are told, was truly grand; the hearse was transformed into a car of triumph; the procession through the streets of Paris occupied upwards of five hours; the great capital was awe-struck, and for a moment dumb with grief. Who can forget the moment when the bier stopped in the Place Vendome under the eyes of that other genius that hovers on the summit of its pillar of brass? There was something sublime in this salutation of the mighty dead!

In the hall of instruction at the Val de Grace, where the statue was erected, three large and decorated tents had been put up for the accommodation of the visitors, one being reserved for the ladies. In the centre was a desk or tribune for the orators who were to address the assembly. Every place was occupied long before the hour appointed for the commencement of the imposing ceremony. Groupes of soldiers were arranged round the tents, and the musicians had taken their seats. At two o'clock precisely, the various members of the commission arrived to the sound of trumpets, headed by M. *Orfila*, the president, in his crimson robes. Among the most conspicuous of the deputation was the noble and illustrious *Larrey*, who seemed to wear on his brow that magnificent appellation, "homme monument," applied to him by the emperor on his death-bed. "The moment of most intense interest has now arrived; anxiety is painted on every countenance; the trumpets are blown; and the veil, which had hitherto concealed the statue, is withdrawn. All the assembly rise at one instant; and, with clapping of hands, exclaim, "Voilà Broussais!" There he is at home, in his own Val de Grace, among his friends, surrounded by his disciples and his children. He is seated in his arm chair, attired in his robe-de-chambre, long trowsers, and slippers, such as we have so often seen him in his study." Our thoughts were involuntarily carried back to the time of our medical studies; we remembered the patient visit of the great physician; we saw him stop at every bed; we heard his voice ever mild and encouraging to his patients—for this man, terrible although he was in intellectual conflicts, this truly formidable dialectician, had his moments of most winning and almost infantine sweetness.

M. *Bouillaud* then mounted the tribune as the representative of the faculty; and never was the inspiration of this ardent professor more lively or more attractive. He was deeply affected; his austere visage wore the expression of a feeling at once sad and triumphant. The recollection of a departed master and friend depressed, while the glory of the apotheosis elevated, his mind.

"Broussais!" he exclaimed, "thy day of judgment hath now come: and when I here solemnly proclaim that you were a man of genius, I feel confident that every listener will respond to my words, and that posterity will confirm their truth. Yes, it was you who laid the edifice of medicine on its proper foundation, and erected it upon the basis of anatomy and physiology. You have shed over the whole domain of our science the rays of the clearest light, by substituting for what you so happily designated 'ontology' the physiological doctrine of medicine, and by completing the work of the great *Bichat.* You have revealed one of the most important truths of medicine by demonstrating that essential fevers are only disguised inflammations; and you have conferred the most signal benefit on humanity by substituting the antiphlogistic for the stimulant and incendiary method of treatment in the cure of those monsters (to borrow your own energetic language) known by the name of ataxic, adynamic, and malignant symptoms. You have revealed another important truth—that diseases are not necessarily subject to the course and duration which have been assigned to them by your predecessors, but that these points in their history are chiefly dependent upon the mode and the energy of the treatment employed. (We suppose that this remark alludes to pyrexiæ in general, and more especially to the groupe of the exanthemata.) In fine, you have created a theory as just as it is ingenious and brilliant, tracing to a process of chronic inflammation a multitude of lesions which have been termed organic, and which before your time were considered as diseases *sui generis*, and independent of all inflammatory action."

Alluding to the fine statue before them, M. *Bouillaud* continued:—"There stands the great reformer of medicine in all the beauty and grandeur of his expression, in all his force, in all his vigour, in all his energy, in all his severity, and, if I may venture to say so, in all his hardy, resolute, and even threatening mien. There stands *Broussais* alive, his head filled with high meditations, with deep thoughts, with revolutionary enterprises; trampling under his Herculean foot those works of ontology on which he had passed so signal a condemnation. His look is bold, imperious, and confident; his mouth is slightly contracted, his upper lip quivering with that peculiar movement which was so characteristic of the great original. Listen, for it seems as if this statue, like that of Memnon, was about to give out an oracular sound." . . . . . . . . "However, true honour to the *Phidias* who has represented so admirably the hero of this solemnity. The statue is truly worthy of the personage, and what can be said more in its praise? There is only one monument that can be more durable and glorious; and that is the works of *Broussais* himself."

When M. *Bouillaud*, pointing to the statue of his master, uttered with his thrilling voice these "grand paroles," the whole assembly was visibly affected with "une emotion immense." The orator sat down among the most rapturous applauses, which drowned for a moment the sound of the trumpets!

